# Is Reorganization Necessary?

**Published:** 1896-10

**Authors:** 


### Editorial.

### IS REORGANIZATION NECESSARY?

The organization of a large body of workers in any field of labor must necessarily be a matter of slow growth. The crudities of the beginning eventually give place to more perfected plans, the result of experience based on an intelligent appreciation of the increased requirements and enlarged spheres of activity. It is safe to assume that nothing originated from the human brain perfect, or is it to be presumed that such a condition is ever reached through our finite efforts. While this is true, it does not follow that intelligent men and women must sit with folded hands and wait for the period of ultimate perfection, if that be possible of attainment. It is immaterial to the thoughtful, progressive mind whether this can ever be accomplished; but every step gained brings humanity to a higher level, and it is to secure this elevation that combination of effort is essential. It is a lamentable fact that the largest portion of the world of sentient human life is indifferent to progress. To these it is sufficient that one day follows another, and year after year remains practically the same.

The activities of any given calling must, therefore, be confined to the few, and discouragement continually awaits these in all their efforts.

The organization of dentistry into society work had its crude beginnings and its martyr period. Those familiar with its history will recall the earnest efforts of Horace H. Hayden to bring the isolated practitioners into one compact body. His repeated efforts and equally repeated failures to accomplish this is pathetic reading in the light of recent history. Those of us who had no part in this cannot appreciate the struggle, and when finally the American Society of Dental Surgeons was organized in New York City, in 1840, with Horace H. Hayden as its first president, it contained so much within it of a disintegrating character that it is not surprising that its life was limited to sixteen years.

One year before its dissolution, in 1855, the American Dental Convention was organized, the second claiming a national character. This organization, in adopting a greater freedom in membership, laid the seeds of its own destruction, and its career closed after a long period of inactivity.

The dissatisfaction occasioned by the methods of the American Dental Convention led in 1859 to the organization of the American Dental Association, which has had now for thirty-seven years uninterrupted prosperity, and has commanded the respect of the dental profession throughout the United States.

Thus, within a period of but little over fifty years, dentists have seen the establishment of three national organizations. In addition to these, we have had the various State societies, the Southern Dental Association, the Section of Dental and Oral Surgery of the American Medical Association, and, more recently, an effort has been made with success to combine several States into a convention, as in the Interstate Meeting held at Excelsior Springs, Mo.

While all this evidences activity, it has a weakening tendency. There is a limit to mental as well as to physical power, and the more this is extended the thinner will become the mental pabulum. Dentistry has not a large field in which to work, and if this be tilled over and over again, the soil will eventually fail to yield anything. It seems, therefore, that the time has arrived in which we must seriously consider whether the energy displayed is not positively injurious to the entire body, and, if so, whether it would not be better to recognize the fact, and at once attempt a consolidation of various interests.

It is, perhaps, hopeless to expect more than a very small minority of the twenty thousand dentists of the United States to take an active part in any organization. The reasons for this are well understood, and while this apathy is lamentable, it must not be permitted to affect associative effort on the profession as a whole.

The time is ripe for a change. This has been made very clear the past year, but exactly what that change is to be is not by any means so evident. The close of the century finds us in the thrall of indifference. Men seem to be losing interest and faith in all forms of effort, and seem ready to let the professional ship drift into the trough of the sea, with no directing head. *This must not be.* The hour of greatest discouragement is the hour for work, and, in the end, port will be reached in safety.

It must be evident to every thinking mind that a profession governed without system will fail to do any good work, and must eventually lapse into a disorganized mass. To prevent this a national organization is a necessity. If we add to this subordinate branches for different sections of the United States, and these finally ending in local societies, the dental profession will be sufficiently organized for an indefinite period.

In a recent article from the pen of Dr. A. H. Thompson, of Topeka, Kan., this subject was clearly stated, and, while we cannot entirely indorse his plan, the spirit of his suggestions meets our warmest commendation. His plan, in brief, is "to have district organizations, which shall be federated under one central organization, but which shall each meet within the limits of their respective districts at times so arranged that they shall not conflict with each other. . . .

"The whole United States can best be divided into four districts,—the Eastern, Southern, Central, and Western. . . . It is noticeable that in three of these districts organizations already exist. . . . In the Atlantic region we have the present American Dental Association, in the Gulf region we have the Southern Association, and in the Pacific region we have the Pacific Coast Association. . . . It would only remain for the profession of the Mississippi basin region to organize a branch to make the system complete."

It is apprehended that while this seems complete on paper, it will be found very difficult to carry out. The power to organize these bodies does not at present belong to any existing society, and there does not seem sufficient enthusiasm in the dental profession to bring such a system into active working order. If it could be arranged as outlined, it would solve the problem of organization, but, it is feared, would increase the weakness to which allusion has been made by multiplying societies. This might, in degree, be obviated by limiting the meeting of branches to once in three years.

The idea entertained by Dr. Thompson of relegating the American Dental Association to an inferior place cannot be entertained. It has occupied the position of a national organization too long and too successfully to expect that the members will be willing to sink its work to that of a subordinate body. It is truly a national society, for its membership at last report represented twenty-nine States, including the District of Columbia and two foreign countries. If any change should ever be contemplated, this organization should hold the pre-eminent position it has justly earned.

The next meeting of this body will take place at Old Point Comfort, Va., and it decided to meet there ostensibly for the purpose of effecting some amicable change in the organization of the two societies,—the Southern and the American. There is probably no dissenting voice as to the necessity for some rearrangement, but the difficulty of effecting this without disturbing deeply rooted attachments seems almost insurmountable. If Dr. Thompson's plan, or something similar, be accepted, the path towards union would seem to be a comparatively easy one. The Southern could then maintain its organization, hallowed by many sacred memories, and continue as the representative body of the Southern States. Those of the members who desire so to do could join the central body, and together work for a systematic organization of the dental profession in its subordinate relations.

The year is before us. It should be a year of thought. Let the matter find its way into our local societies, and be there discussed. Awaken the members from the sleep of years to their just responsibilities as individuals. Instil into their minds the one important idea that every man and woman belonging to the profession has a duty to perform, and that this lies in the direction of repaying, in part, that which he or she has received through the work of others, and by this effort help to make our organization worthy of the progressive age in which we live.

